# Prognosis After Seven Days of Veno‐Venous Extracorporeal Membrane Oxygenation Support

**DOI:** 10.1111/aor.15032

**Published:** 2025-06-03

**Authors:** Felix A. Rottmann, Rebecca Book, Alexander Supady, Viviane Zotzmann, Markus Jäckel, Alexander Maier, Frederic Arnold, Dirk Westermann, Tobias Wengenmayer, Jonathan Rilinger, Dawid L. Staudacher

**Affiliations:** ^1^ Department of Medicine IV – Nephrology and Primary Care, Medical Center and Faculty of Medicine University of Freiburg Freiburg Germany; ^2^ Interdisciplinary Medical Intensive Care, Medical Center and Faculty of Medicine University of Freiburg Freiburg Germany; ^3^ Department of Cardiology, Pneumology, Angiology, Intensive Care and Thoracic Surgery Ortenau Clinical Center Offenburg‐Kehl Offenburg Germany; ^4^ Department of Cardiology and Angiology University Heart Center Freiburg – Bad Krozingen and Faculty of Medicine, University of Freiburg Freiburg Germany; ^5^ Institute for Microbiology and Hygiene, Medical Center and Faculty of Medicine University of Freiburg Freiburg Germany

**Keywords:** ARDS, ECMO, extracorporeal life support, prognosis, prognostication, survival

## Abstract

**Background:**

Prognostication in patients receiving veno‐venous extracorporeal membrane oxygenation (V‐V ECMO) remains challenging, particularly during prolonged support. Accurate prognostic indicators are essential for patients, caregivers, and clinicians. This study evaluates outcomes in patients supported with V‐V ECMO for at least seven days.

**Methods:**

We conducted a single‐center retrospective cohort study of patients cannulated for V‐V ECMO due to respiratory failure. The primary endpoint was hospital survival. The subgroup of patients still on V‐V ECMO on day 7, stratified by respirator and ECMO parameters, was analyzed with respect to predictors of the primary outcome. A survival favorability margin was defined as the bound of the hospital survivors' 95% confidence interval closest to the non‐survivor median.

**Results:**

Among 299 patients treated with V‐V ECMO (median age 55 years, 66.9% male), hospital survival was 44.8%. Pneumonia was the primary cause of respiratory failure in 216 patients (72.2%). By day 7, 182/299 patients (60.9%) remained on V‐V ECMO, with a hospital survival rate of 45.1%. Three respiratory parameters (ventilator FiO_2_, tidal volume, compliance) and three V‐V ECMO parameters (blood flow, sweep gas flow, O_2_ fraction) significantly predicted hospital survival (all *p* ≤ 0.015). All six predictors separated survivors and non‐survivors through day 1 to 10 (all *p* < 0.001). Moreover, the clustering of several parameters above the favorability margin showed a clear discrimination of the overall survival (≥ 5 vs. zero parameters, survival 67.5% vs. 13.8%, OR 13.0, 95% CI 3.5–38.6, *p* < 0.001).

**Conclusion:**

Among patients still on V‐V ECMO on day seven, respiratory and ECMO parameters above the favorability margin correlated strongly with hospital survival. Survival was rare in patients with all predictors below the favorability margin. Day seven could provide a useful, though not definitive, time point for prognostication based on respiratory and ECMO parameters.

## Graphical abstract

The hospital survival rate of patients still on V‐V ECMO after seven days was 45.1%. Survival correlates with the respiratory situation on day seven defined as points scored above the favorability margin of survival. Day 7 may be too early to predict prognosis in V‐V ECMO.

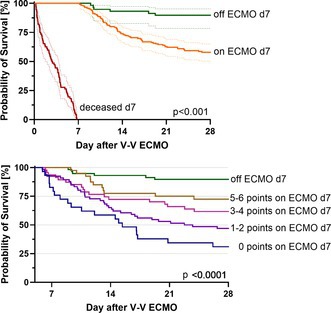

## 1 Introduction

Respiratory failure due to acute respiratory distress syndrome (ARDS) occurs in one out of ten critically ill patients [1]. The mortality rate is 40%–50% [2, 3]. Evidence‐based management of patients with ARDS includes treatment of the underlying pathology (like pneumonia or sepsis), respiratory support, fluid management, and supportive measures [4, 5].

In severe ARDS, veno‐venous extracorporeal membrane oxygenation (V‐V ECMO) has been suggested to improve survival [6, 7, 8]. But even in those patients with optimal management, mortality in the ECMO to Rescue Lung Injury in Severe ARDS (EOLIA) study was as high as 35% [6]. Several studies have tried to define prognostic scoring systems for these patients, including the Respiratory Extracorporeal Membrane Oxygenation Survival Prediction (RESP) [9] and the PRedicting dEath for SEvere ARDS on VV‐ECMO (PRESERVE) score [10]. Both scores factor in parameters at V‐V ECMO initiation to estimate survival but cannot be used for V‐V ECMO indication. Overall predictive value might be mediocre [11].

After V‐V ECMO cannulation for ARDS, the intensive care unit (ICU) course is prolonged and unpredictable. In the EOLIA study, patients were on the ICU for a median of 23 days with a median hospital stay of 36 days [6]. A prolonged treatment period with invasive, resource‐intensive ECMO support requires continuous reevaluations of the patient's wishes and an alignment of these wishes with prognosis [12]. Especially in those without options for lung transplantation, a bridge‐to‐nowhere scenario can be ethically challenging to patients, relatives, and caregivers alike [13, 14].

In patients without respiratory improvement after one week of V‐V ECMO support, the indication for continuation may be questionable. However, limited data exist on this patient population. We therefore retrospectively analyzed a single‐center registry of patients receiving V‐V ECMO for seven days, with or without respiratory improvement, to assess outcomes. We hypothesized that patients without improvement might still have a considerable chance of survival, thus justifying prolonged V‐V ECMO therapy. The primary outcome was hospital survival.

## 2 Methods

### 2.1 Registry Setup and Data Acquisition

All patients cannulated for V‐V ECMO therapy between January 2009 and April 2020 at the Interdisciplinary Medical Intensive Care (IMIT) unit of the Medical Center of the University of Freiburg, Germany were included in this registry and analyzed via a retrospective cohort study. The limitation for data acquisition in April 2020 was chosen as the beginning of the COVID‐19 pandemic in Germany. The ethics committee of the University of Freiburg (file number EK‐Freiburg 553/19) approved this registry. Informed consent was waived by the ethics committee. Data was checked for plausibility according to the RECORD statement [15] and is presented according to the STROBE guidelines [16]. Inclusion criteria were V‐V ECMO support indicated on current guidelines [17], admission for respiratory failure due to ARDS based on the Berlin definition [3] and an age of > 18 years. Patient identification began with a computerized search for the OPS (German operation and procedure classification system) code for ECMO (8–852). The other criteria were then applied on a case‐by‐case basis when analyzing individual patient files. Data was collected partly computerized using the electronic patient files. In addition, each file and discharge letter were revised manually.

Primary endpoint was hospital survival. Thus, patients were categorized into two groups as hospital survivors and deceased. Baseline parameters and the general characteristics of the ICU stay were collected as presented in Tables 1 and 2. Data on oxygenation via ventilator (e.g., positive end expiratory pressure (PEEP), inspiratory oxygen fraction (FiO_2_)) and ventilation (e.g., tidal volume and compliance) as well as V‐V ECMO parameters (i.e., blood flow, sweep gas flow and O_2_ fraction) were collected daily for the first 10 days after implantation of the first V‐V ECMO. Definitions were as follows: Average values of all measurements of individual calendar days were used to represent those days for each patient. Time on V‐V ECMO was measured from implantation of the first device to either successful decannulation for more than 48 h or death. The predefined use of calendar days accounts for the first day being < 24 h.

**TABLE 1:** Baseline characteristics by hospital survival.

Baseline characteristics	Total (*n* = 299)	Survived (*n* = 134)	Deceased (*n* = 165)	*p*
Percentage of patients [%]	100	44.8	55.2	
Age [years]	55 (44–64)	53 (42–60)	57 (46–67)	**0.021** ^**a**^
Male gender	200 (66.9%)	92 (68.7%)	108 (65.5%)	0.622^b^
BMI [kg/m^2^]	24.5 (23.5–29.4)	24.5 (22.9–30.4)	24.7 (23.6–28.1)	0.639^a^
Preexisting conditions				
Pulmonary disease (any)	88 (29.4%)	33 (24.6%)	55 (33.3%)	0.126^b^
COPD	25 (8.4%)	11 (8.2%)	14 (8.5%)	0.999^b^
Asthma	17 (5.7%)	8 (6.0%)	9 (5.5%)	0.999^b^
Pulmonary fibrosis	26 (8.7%)	2 (1.5%)	24 (14.5%)	**0.001** ^**b**^
Pulmonary hypertension	8 (2.7%)	1 (0.7%)	7 (4.2%)	0.078^b^
Long term oxygen therapy	14 (4.7%)	3 (2.2%)	11 (6.7%)	0.098^b^
Cystic fibrosis	7 (2.3%)	1 (0.7%)	6 (3.6%)	0.135^b^
Nicotine use disorder	99 (33.1%)	50 (37.3%)	49 (29.7%)	0.176^b^
Hypertension	102 (34.1%)	50 (37.3%)	52 (31.5%)	0.327^b^
Coronary artery disease	36 (12.0%)	13 (9.7%)	23 (13.9%)	0.288^b^
Chronic kidney disease	21 (7.0%)	8 (6.0%)	13 (7.9%)	0.651^b^
CKD with regular dialysis	2 (9.5% of CKD)	1 (12.5% of CKD)	1 (7.7% of CKD)	0.999^b^
Chronic liver disease	22 (7.4%)	4 (3.0%)	18 (10.9%)	**0.013** ^**b**^
Diabetes mellitus	39 (13.0%)	17 (12.7%)	22 (13.3%)	0.999^b^
Immunosuppression	90 (30.1%)	25 (18.7%)	65 (39.4%)	**0.001** ^**b**^
Cause of respiratory failure				
Pneumonia	216 (72.2%)	94 (70.1%)	122 (73.9%)	
Covid‐19	10 (4.6% of P.)	5 (5.3% of P.)	5 (4.1% of P.)	
Aspiration	25 (8.4%)	10 (7.5%)	15 (9.1%)	
Other	58 (19.4%)	30 (22.4%)	28 (17.0%)	0.472^c^
Situation before ECMO‐initiation				
Horowitz index pre‐implant (*n* = 272:115:157)	73.1 (60.9–97.1)	77.0 (62.0–106)	70 (59.5–96)	0.250^a^
Duration of MV before ECMO [d] (*n* = 280:127:153)	1.2 (0.3–3.4)	1.1 (0.3–3.1)	1.4 (0.3–5.3)	0.424^a^
Prone positioning before ECMO	72 (24.1%)	31 (23.1%)	41 (24.8%)	0.786^b^
Cardiac arrest before ECMO (*n* = 295:131:164)	40 (13.4%)	15 (11.2%)	25 (15.2%)	0.394^b^
SOFA score pre‐implant	13 (10–15)	12 (10–15)	13 (10–16)	0.431^a^
APACHE‐II score pre‐implant	26 (21–32)	25 (19–31)	27 (21.5–33)	**0.037** ^**a**^
RESP score pre‐implant	1 (−1 to 3)	2 (0–4)	1 (−2 to 3)	**0.006** ^**a**^

*Note:* Data given in median (interquartile range) or in number of patients (percentage of group). *p*‐values were calculated between groups using either ^a^Mann–Whitney‐*U* test, ^b^Fishers Exact test or ^c^Chi‐squared test. The *p*‐value is reported in bold if the differences are statistically significant (*p* < 0.05). Number of values indicated if *n* < 299.

Abbreviations: BMI, body‐mass‐index; CKD, chronic kidney disease; COPD, chronic obstructive pulmonary disease; ECMO, extracorporeal membrane oxygenation; MV, mechanical ventilation; P., pneumonia.

**TABLE 2:** Treatment characteristics and endpoints by hospital survival.

	Total (*n* = 299)	Survived (*n* = 134)	Deceased (*n* = 165)	*p*
General ICU treatment characteristics				
ICU length of stay [d]	13.6 (9–23.8)	18 (11.8–32.9)	11.2 (5.6–19.0)	**< 0.001** ^**a**^
MV duration [d] (298:134:164)	13 (7.8–23.1)	14.9 (9.6–30.9)	11.2 (5.9–19.7)	**< 0.001** ^**a**^
MV duration under ECMO [d]	10 (5.5–18.8)	12.9 (8.4–24.7)	8.8 (3.2–14.8)	**< 0.001** ^**a**^
Prone positioning during ECMO	80 (26.8%)	32 (23.9%)	48 (29.1%)	0.358^b^
Tracheotomy	115 (38.5%)	65 (48.5%)	50 (30.3%)	**0.002** ^**b**^
Acute dialysis	114 (38.1%)	49 (36.6%)	65 (39.4%)	0.634^b^
ECMO treatment characteristics				
Inhouse Implantation	224 (74.9%)	88 (65.7%)	136 (82.4%)	**0.001** ^**b**^
Dual lumen cannulation	250 (83.6%)	116 (86.6%)	134 (81.2%)	0.272^b^
ECMO runtime [d]	6.8 (4–12.6)	6.7 (4.5–11.9)	7.2 (3.3–13.4)	0.914^a^
Time from ECMO implant to discharge or death [d]	12.1 (6.3–22.6)	17.2 (10.7–31.3)	9.1 (3.7–15.1)	**< 0.001** ^**a**^
Awake ECMO therapy	18 (6.0%)	6 (4.5%)	12 (7.3%)	0.341^b^
Weaning successful	158 (52.8%)	134 (100%)	24 (14.5%)	**0.001** ^**b**^
30‐d survival	147 (49.2%)	133 (99.3%)	14 (8.5%)	**0.001** ^**b**^

*Note:* Data given in median (interquartile range) or in number of patients (percentage of group). *p*‐values were calculated between groups using either ^a^Mann–Whitney *U* test or ^b^Fishers Exact test. The *p*‐value is reported in bold if the differences are statistically significant (*p* < 0.05). Number of values indicated if *n* < 299.

Abbreviations: ECMO, extracorporeal membrane oxygenation; ICU, intensive care unit; MV, mechanical ventilation.

### 2.2 Local ECMO Center Standards

The IMIT, located at the University Hospital Freiburg, offers 24/7 ECMO for both, veno‐venous and veno‐arterial support. As previously described, respiratory support is primarily achieved by dual‐lumen cannulation of the right internal jugular vein [18] for both in‐house and retrieval cases [19]. After V‐V ECMO initiation, our local standard includes early prone positioning combined with “baby‐lung” ventilation to minimize ventilator‐induced lung injury. Typical ventilator settings on V‐V ECMO are: PEEP of 15 cm H_2_O, a plateau pressure of 25 cm H_2_O, a FiO_2_ of 50%, and a respiratory rate of 10 breaths per minute [20].

### 2.3 V‐V ECMO Center Weaning Standards

During V‐V ECMO support, lung‐protective ventilation needs to be provided [21]. Specifically, we aim for a ventilator FiO_2_ of ≤ 0.5, PEEP ≤ 15 mbar, and tidal volumes of 4–6 mL/kg ideal body weight, with a driving pressure of ≤ 15 mbar and a plateau pressure ≤ 30 mbar, while maintaining paO_2_ > 60 mmHg and paCO_2_ at 35–45 mmHg. With lung‐protective ventilation and improvement in pulmonary function, our protocol advocates weaning from V‐V ECMO before weaning from mechanical ventilation. In improved oxygenation, V‐V ECMO blood flow is reduced to 3 L/min before decreasing the sweep gas O_2_ fraction. In improved ventilation, V‐V ECMO sweep gas flow is weaned. Before V‐V ECMO explantation, a minimum 6‐h trial with 0 L/min sweep gas flow is required. Final therapeutic decisions are made on a case‐by‐case basis at the bedside, considering each patient's individual condition.

### 2.4 Statistical Analysis

Pooled data as described above was analyzed. Continuous data was tested for normality using D'Agostino & Pearson test showing none of the data was normally distributed. We then performed Mann–Whitney‐*U* tests. Fisher's Exact test and Chi‐squared test were used for the analysis of categorical data. Data are given in median (interquartile range) or in number of patients (percentage of group).

To compare the development of the various parameters of ventilator and V‐V ECMO settings over the first 10 days in the groups of hospital survivors and deceased, we then performed 2‐way ANOVAs. As post hoc tests to compare groups on individual days, we performed Šídák's multiple comparisons test.

Day seven landmark analysis of surrogates of hospital survival: predefined plausible predictors of the primary endpoint (ventilator FiO_2_, PEEP, plateau pressure, tidal volume, respiratory rate, compliance, ECMO blood flow, sweep gas flow, and O_2_ fraction) were tested using univariate logistic regression. Subsequently, for all predictors mean values and 95% confidence intervals of survivors still on ECMO on day seven were calculated. The favorability margin was defined as the 95% CI bound of hospital survivors still on ECMO on day 7 closest to the median of non‐survivors. Points were scored for values above the favorability margin where higher values indicate a positive outcome (e.g., tidal volume). Moreover, points were scored for values below the favorability margin where lower values indicate a positive outcome (e.g., ventilator FiO_2_). Patients were stratified based on predictor values beyond this margin for subgroup comparisons where more points scored indicated better respiratory performance or less (ventilator or ECMO) support.

A Kaplan–Meier survival curve was created to visualize these subgroups. The analysis included patients with a follow‐up to hospital discharge of less than 28 days. These patients were treated as surviving until day 28 even though information on events after hospital discharge was not available in our dataset. To compare our model of choice to either an earlier decision based on the confidence interval of surviving patients still on ECMO on day 4 or to using the mean instead of the 95% CI as the cutoff, we created receiver operating characteristic curves calculating the area under the curve (AUC). *p*‐values < 0.05 were considered statistically significant. As statistical software, we used GraphPad Prism (Version 10, GraphPad, San Diego, CA, USA) and IBM SPSS Statistics (Version 29, IBM Statistics, Armonk, NY, USA).

## 3 Results

### 3.1 Baseline

Two hundred and ninety‐nine patients required V‐V ECMO support between January 2009 and April 2020. The median age was 55 (44–64) years and 66.9% were male (Table 1). Median body mass index was 24.5 (23.5–29.4) kg/m^2^ and 88/299 (29.4%) were cannulated for V‐V ECMO with a preexisting pulmonary disease. A total of 26/299 (8.7%) patients suffered from pulmonary fibrosis, a condition significantly more common in deceased patients compared to hospital survivors (*p* = 0.001). The cause of respiratory failure was pneumonia in 216/299 (72.2%), followed by aspiration in 25/299 (8.4%) and other in 58/299 (19.4%). Median Horowitz index pre‐cannulation was 73.1 (60.9–97.1) not differing between survivors and non‐survivors (*p* = 0.250). Duration of mechanical ventilation before V‐V ECMO therapy was similar in both groups at a median of 1.2 (0.3–3.4) days (*p* = 0.424). RESP scores [9] differed significantly at baseline (*p* = 0.006) but median absolute values were almost identical at 2 (0–4) points in hospital survivors and 1 (−2 to 3) point in deceased patients considering that both are classified as risk class III (−1 to 2 points) with an expected hospital survival rate of 57%.

### 3.2 General ICU Treatment Characteristics

Hospital survivors had significantly longer ICU stays compared to deceased patients and required a longer duration of mechanical ventilation (both *p* < 0.001, respectively). Prone positioning during V‐V ECMO was performed in 80/299 (26.8%) similarly in survivors and deceased patients (*p* = 0.358). Median V‐V ECMO runtime was 6.8 (4–12.6) days. In‐house V‐V ECMO implantation was more frequent among deceased patients (136/165, 82.4%) compared to hospital survivors (88/134, 65.7%) (*p* = 0.001). For details see Table 2.

### 3.3 Respiratory and ECMO Settings Day 1 to 10

**FIGURE 1:**
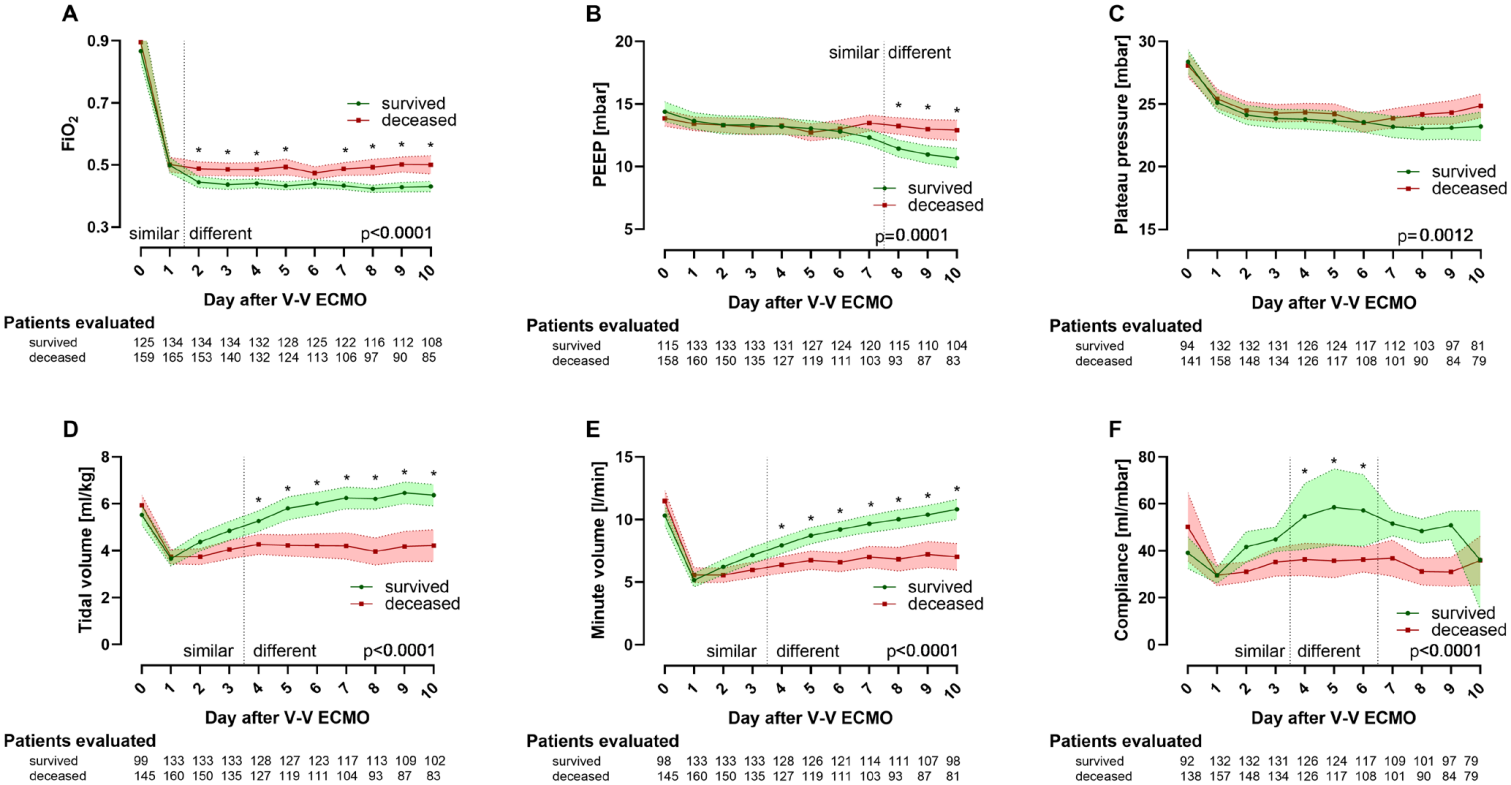
Ventilator settings in ARDS including all patients (i.e., those already weaned from V‐V ECMO). All patients started V‐V ECMO therapy on day 0. P_group_ is shown in each figure. (A) Ventilator FiO_2_, (B) Ventilator PEEP, (C) Ventilator plateau pressure, (D) Tidal volumes, (E) Minute volumes, (F) Compliance. * marks significant post hoc tests comparing groups on individual days. FiO_2_, inspiratory oxygen fraction; PEEP, positive end‐expiratory pressure; V‐V ECMO, Veno‐venous extracorporeal membrane oxygenation. [Color figure can be viewed at wileyonlinelibrary.com]

After initiation of V‐V ECMO, FiO_2_ and tidal volumes were reduced. During the first 10 days, hospital survivors then had significantly lower ventilator FiO_2_ and PEEP levels and significantly higher tidal volumes, minute volumes, as well as pulmonary compliance compared to hospital non‐survivors (all *p*_group_ < 0.0001, Figure 1). However, the separation of the two groups occurred at different time points, with FiO_2_ being the earliest (day 2, Figure 1). Plateau pressure was similar in hospital survivors and non‐survivors over all 10 days. When the analysis was restricted to patients actually on V‐V ECMO support (censoring patients after ECMO weaning), the results were consistent (Figure S1).

**FIGURE 2:**
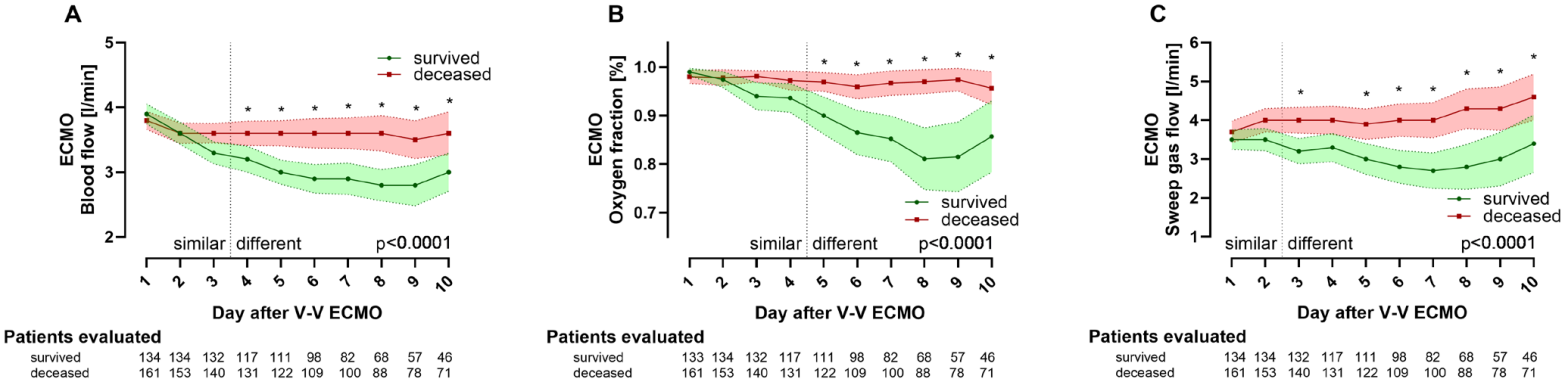
V‐V ECMO settings in ARDS. All patients started V‐V ECMO therapy on day 0. P_group_ is shown in each figure. (A) ECMO blood flow, (B) ECMO oxygen fraction, (C) ECMO sweep gas flow. * marks significant post hoc tests comparing groups on individual days. V‐V ECMO, Veno‐venous extracorporeal membrane oxygenation. [Color figure can be viewed at wileyonlinelibrary.com]

Hospital survivors also had significantly lower V‐V ECMO blood and sweep gas flow, and lower ECMO oxygen fraction during the first 10 days after cannulation (all *p*_group_ < 0.0001, Figure 2). Here, sweep gas flow was first to differ between.

Of note, ventilator driving pressure was reduced immediately after ECMO initiation in both groups (*p*_time_ < 0.0001) and did not differ significantly between survivors and non‐survivors (*p*_group_ = 0.0796, Figure S2).

### 3.4 Landmark Analysis: Patients Still on V‐V ECMO Day 7

One hundred eighty‐two out of 299 patients were still on V‐V ECMO on day 7 (including 82/182 hospital survivors). In those patients, univariate logistic regression analysis identified ventilator FiO_2_, tidal volume, compliance, V‐V ECMO blood, sweep gas flow, and V‐V ECMO oxygen fraction as predictors of hospital survival (all *p* < 0.015, Table 3). As predefined, a favorability margin was determined for each of these parameters (Table 3). Patients with 5–6 parameters above the favorability margin had a hospital survival of 27/40 (67.5%) compared to 4/29 (13.8%) in patients with zero parameters above the margin (*p* < 0.0001, OR of 13.0 (3.5–38.6)). For all parameters see Table S2.

**TABLE 3:** Univariate logistic regression analysis on hospital survival of all patients still on V‐V ECMO on day 7.

Variable	Univariate logistic regression analysis (*n* = 182)			Survival predictors (*n* = 82/182)		
	Odds ratio	95% confidence interval	*p*	Favorability margin	Occurrence	Percentage
d7 Ventilator FiO_2_	0.003	0.000–0.132	**0.003**	≤ 0.46	118/182	64.8%
d7 PEEP	1.014	0.918–1.121	0.779			
d7 Plateau pressure	0.998	0.928–1.073	0.960			
d7 Tidal volume	1.255	1.111–1.418	**0.001**	≥ 5.0 mL/kg BW	73/182	40.1%
d7 Respiratory rate	0.989	0.951–1.028	0.581			
d7 Compliance	1.014	1.003–1.026	**0.015**	≥ 44.0 mL/mbar	63/182	34.6%
d7 ECMO blood flow	0.578	0.438–0.762	**0.001**	≤ 3.1 L/min	90/182	49.5%
d7 ECMO sweep gas flow	0.773	0.670–0.892	**0.001**	≤ 3.2 L/min	93/182	51.1%
d7 ECMO oxygen fraction	0.960	0.940–0.980	**0.001**	≤ 0.90	34/182	18.7%

*Note:* Potential predictors of the primary endpoint (hospital survival) were tested in a univariate logistic regression analysis. The *p*‐value is reported in bold if the differences are statistically significant (*p* < 0.05). Absolute values were used for calculations. Only predictors of survival in the univariate logistic regression analysis were used in the survival model. Scoring from 0 to 6 points in the survival model was based on patients with better respiratory settings than the more morbid bound of the 95% CI of the 82 surviving patients. For complete descriptive statistics of all 82 surviving patients see Table S1.

Abbreviations: BW, body weight; CI, confidence interval; d, day; ECMO, extracorporeal membrane oxygenation; FiO_2_, inspiratory oxygen fraction; PEEP, positive end‐expiratory pressure.

**FIGURE 3:**
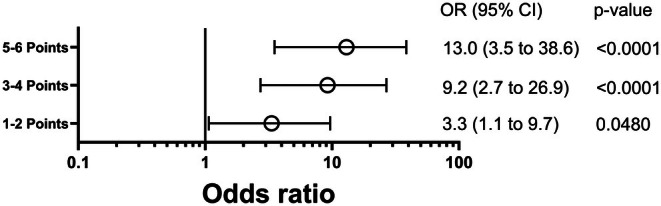
Odds ratio of hospital survival in V‐V ECMO according to points scored above the favorability margin. Each point value was compared to patients with 0 points, and an OR for each outcome was calculated. An odds ratio > 1 indicates a better outcome. CI, Confidence interval; OR, Odds ratio.

**FIGURE 4:**
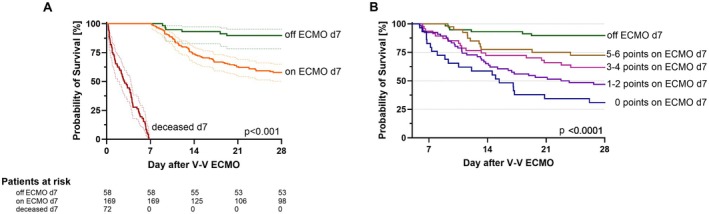
Kaplan–Meier analysis of 28‐day survival of patients still on V‐V ECMO on day 7. (A) Landmark analysis comparing patients still on V‐V ECMO on day 7 (orange) to those already weaned from ECMO (green). Patients already deceased on day 7 are shown as reference (red). (B) Landmark analysis on day 7 comparing different point values in patients still on V‐V ECMO to patients already weaned from ECMO. Censoring before day 7 is due to counting calendar days for all analyses resulting in day 1 being < 24 h. Details are explained in the methods section. d, day; V‐V ECMO, Veno‐venous extracorporeal membrane oxygenation. [Color figure can be viewed at wileyonlinelibrary.com]

Figure 3 shows the ORs of survival compared to patients with zero parameters above the favorability margin. Kaplan–Meier survival analysis of the first 28 days after cannulation for V‐V ECMO highlights significantly higher survival rates in patients already decannulated from ECMO on day 7 (Figure 4A). Points scored above the favorability margin in each of the six predictors of hospital survival further characterize the subgroup of patients still on ECMO on day 7 (Figure 4B).

When comparing this model to either an earlier choice based on the 95% CIs of surviving patients still on ECMO on day 4 (AUC = 0.6525) or a model using the mean of surviving patients still on ECMO on day 7 (AUC = 0.6997) our model performed best with an AUC of 0.7282; see Figure S3.

## 4 Discussion

In this retrospective registry of patients supported with V‐V ECMO, those still on V‐V ECMO on day 7 had an overall hospital survival rate of 45.1%. While hospital survivors had less‐invasive respiratory and V‐V ECMO settings, even those with relatively invasive settings still reached a hospital survival rate of 13.8%.

Mortality in V‐V ECMO for respiratory failure is not linear over the course of therapy. However, unlike patients in shock, most patients do not die early within the first days after cannulation. In the CESAR trial, the median duration of ICU treatment in V‐V ECMO non‐survivors was 11.0 days [7]. In the EOLIA trial, survival in the V‐V ECMO arm was over 80% on day 20 (compared to 54% on day 90). Real‐world data, however, shows that V‐V ECMO patients are usually treated shorter. Even though the best survival is seen in patients for whom ECMO support can be discontinued within one week, still a considerable number of patients survive even after longer V‐V ECMO support durations of over 3 weeks [22]. Prolonged ECMO support, however, presents significant challenges for patients, relatives, and caregivers. When no recovery is perceived, the motivation to continue treatment can decline, particularly given the uncertainty of outcomes, as a lack of recovery might be perceived as a sign of futility. However, data including—ours might—indicate that sustained V‐V ECMO support can still yield positive outcomes [22, 23, 24, 25, 26]. This evidence may help to encourage continued treatment and foster greater confidence among all parties involved.

In our V‐V ECMO cohort on day 7, survivors consistently were treated with less invasive mechanical ventilation settings and required less V‐V ECMO support compared to non‐survivors. Since ventilator settings are systematically adjusted to match lung performance and minimize ventilator‐induced lung injury, this reduction may indicate lung recovery. However, we cannot completely exclude the possibility of treatment bias, as patients with a perceived poor prognosis may not have undergone the same systematic titration. Although standardized protocols are available at our center, adherence cannot be proven retrospectively. More invasive ventilation may contribute to ventilator‐ and patient self‐inflicted lung injury (VILI and P‐SILI, respectively) [27, 28], thereby negatively impacting prognosis. This potential confounder may complicate the interpretation of our findings. However, given the high awareness of P‐SILI at our ARDS center, it seems unlikely that ventilator‐induced lung injury is systematically present in a significant number of patients. Also, in a recent study of more than 600 COVID‐19 ARDS patients, 73% of patients died from non‐pulmonary failure [29], a number similar to the CESAR trial [7].

Our landmark analysis from day 7 of V‐V ECMO offers a distinct advantage over scoring systems that rely solely on baseline cannulation parameters. While traditional scores like SOFA [30], APACHE II [31], or RESP [9] capture a static snapshot, our day 7 measurements reflect the evolving clinical trajectory and patient response to therapy. This more dynamic assessment provides a refined risk stratification and may offer a better prediction of outcomes. Consequently, incorporating day 7 data into prognostic models might support decision‐making and help sustain motivation among patients, families, and caregivers despite delayed early improvement. Nevertheless, these results should be regarded as hypothesis‐generating, as they have not yet been re‐evaluated in an external collective.

## 5 Conclusion

Hospital survival was 44.8% in all patients cannulated with V‐V ECMO for respiratory failure. Among patients still on V‐V ECMO on day 7, ventilator FiO_2_, tidal volume, compliance, V‐V ECMO blood and sweep gas flow, and V‐V ECMO oxygen fraction were identified as predictors of hospital survival. Respiratory and ECMO parameters above the favorability margin correlated strongly with hospital survival. Day 7 could serve as a potential time point for prognostic assessment in V‐V ECMO for respiratory failure, as patients with zero points scored above the favorability margin showed a survival rate of only 13.8% (4/29 patients).

## Author Contributions

Concept/design: Felix A. Rottmann, Jonathan Rilinger, Dawid L. Staudacher; Data analysis/interpretation: Felix A. Rottmann, Rebecca Book, Alexander Supady, Viviane Zotzmann, Markus Jäckel, Alexander Maier, Frederic Arnold, Dirk Westermann, Tobias Wengenmayer, Jonathan Rilinger, Dawid L. Staudacher; Drafting article: Felix A. Rottmann, Jonathan Rilinger, Dawid L. Staudacher; Critical revision of article: Felix A. Rottmann, Rebecca Book, Alexander Supady, Viviane Zotzmann, Markus Jäckel, Alexander Maier, Frederic Arnold, Dirk Westermann, Tobias Wengenmayer, Jonathan Rilinger, Dawid L. Staudacher; Approval of article: Felix A. Rottmann, Rebecca Book, Alexander Supady, Viviane Zotzmann, Markus Jäckel, Alexander Maier, Frederic Arnold, Dirk Westermann, Tobias Wengenmayer, Jonathan Rilinger, Dawid L. Staudacher; Statistics: Felix A. Rottmann, Jonathan Rilinger, Dawid L. Staudacher; Funding secured: Jonathan Rilinger, Dawid L. Staudacher; Data collection: Rebecca Book.

## Conflicts of Interest

F.A.R., R.B., M.J., A.M., and D.W. declare no conflicts of interest. F.A. declares research funding from the Research Commission, Faculty of Medicine, University of Freiburg. V.Z. received travel support from Orion Pharma and lecture honoraria from Medela and AstraZeneca. A.S. declares a research grant, lecture honoraria, and travel support from CytoSorbents Europe, lecture honoraria from AstraZeneca and Getinge, and travel support from Artcline. T.W. received lecture honoraria or travel support from Abbott Medical, AstraZeneca, and Boston Scientific. J.R. received lecture honoraria or travel support from Novartis and AstraZeneca. D.L.S. received lecture honoraria or travel support from Abiomed, AstraZeneca, Dahlhausen, Getinge, Medtronic, Orion Pharma, and was part of a dual‐lumen advisory board by Medtronic.

## Supporting information


Data S1.
