# Supplementary material for: Prognosis After Seven Days of Veno‐Venous Extracorporeal Membrane Oxygenation Support
Source: Artif Organs. 2025 Jun 3;49(10):1579–87. doi: 10.1111/aor.15032 (PMC12625483; doi:10.1111/aor.15032)
Supplement: Supplementary file 1 — Data S1. [file AOR-49-1579-s001.docx]

Supplement

|  | Ventilator FiO_2_ | TV/kg BW [ml] | Compliance [ml/mbar] | ECMO blood flow [l/min] | ECMO sweep gas flow [l/min] | ECMO oxygen fraction |
| --- | --- | --- | --- | --- | --- | --- |
| Number of values | 82 | 78 | 78 | 82 | 82 | 82 |
| Mean | 0.445 | 5.558 | 50.41 | 2.869 | 2.682 | 0.852 |
| Std. Deviation | 0.070 | 2.414 | 28.32 | 1.092 | 2.143 | 0.216 |
| Std. Error of Mean | 0.008 | 0.2734 | 3.21 | 0.1206 | 0.2366 | 0.238 |
| Lower 95% CI of mean | 0.430 | **5.013** | **44.03** | 2.629 | 2.211 | 0.804 |
| Upper 95% CI of mean | **0.461** | 6.102 | 56.80 | **3.109** | **3.153** | **0.899** |

**Suppl. table 1: Hospital survivors still on V-V ECMO on day 7, n=82.**

Descriptive statistics of predictors of the primary endpoint hospital survival are shown. Values used as favorability margin (i.e. cutoff) for scoring points are reported in bold. For example, a ventilator FiO_2_ below 0.46, a TV/kg BW above 5.013 ml and a compliance above 44.03 ml/mbar resulted in scoring one point each as these were defined as the favorability margin of hospital survival. In ECMO blood and sweep gas flow as well oxygen fraction lower values than the favorability margin also resulted in scoring 1 point each. I.e., patients still on ECMO on day 7 could score from 0/6 to 6/6 points. Abbreviations: FiO_2_ = inspiratory oxygen fraction, TV = tidal volume, BW = body weight, V-V ECMO = Veno-venous extracorporeal membrane oxygenation, Std. = Standard, CI = Confidence interval

|  | Total | Hospital survival | Deceased |
| --- | --- | --- | --- |
| 5-6 points | 40 (22.0%) | 27 (67.5%) | 13 (32.5%) |
| 3-4 points | 47 (25.8%) | 28 (59.6%) | 19 (40.4%) |
| 1-2 points | 66 (36.3%) | 23 (34.8%) | 43 (65.2%) |
| 0 points | 29 (15.9%) | 4 (13.8%) | 25 (86.2%) |

**Suppl. Table 2: Scoring of all patients still on V-V ECMO on day 7.** Data given as absolute values. Total n=182. Points could be scored for low ventilator FiO_2_, high tidal volume, high compliance, low ECMO blood flow, low ECMO sweep gas flow and low ECMO FiO_2_. Points were allocated for better respiratory settings than the favorability margin of survival as explained in suppl. table 1. Percentages add up to 100% in the total column and when adding hospital survival and deceased in each row. Abbreviations: FiO_2_ = inspiratory oxygen fraction, V-V ECMO = Veno-venous extracorporeal membrane oxygenation


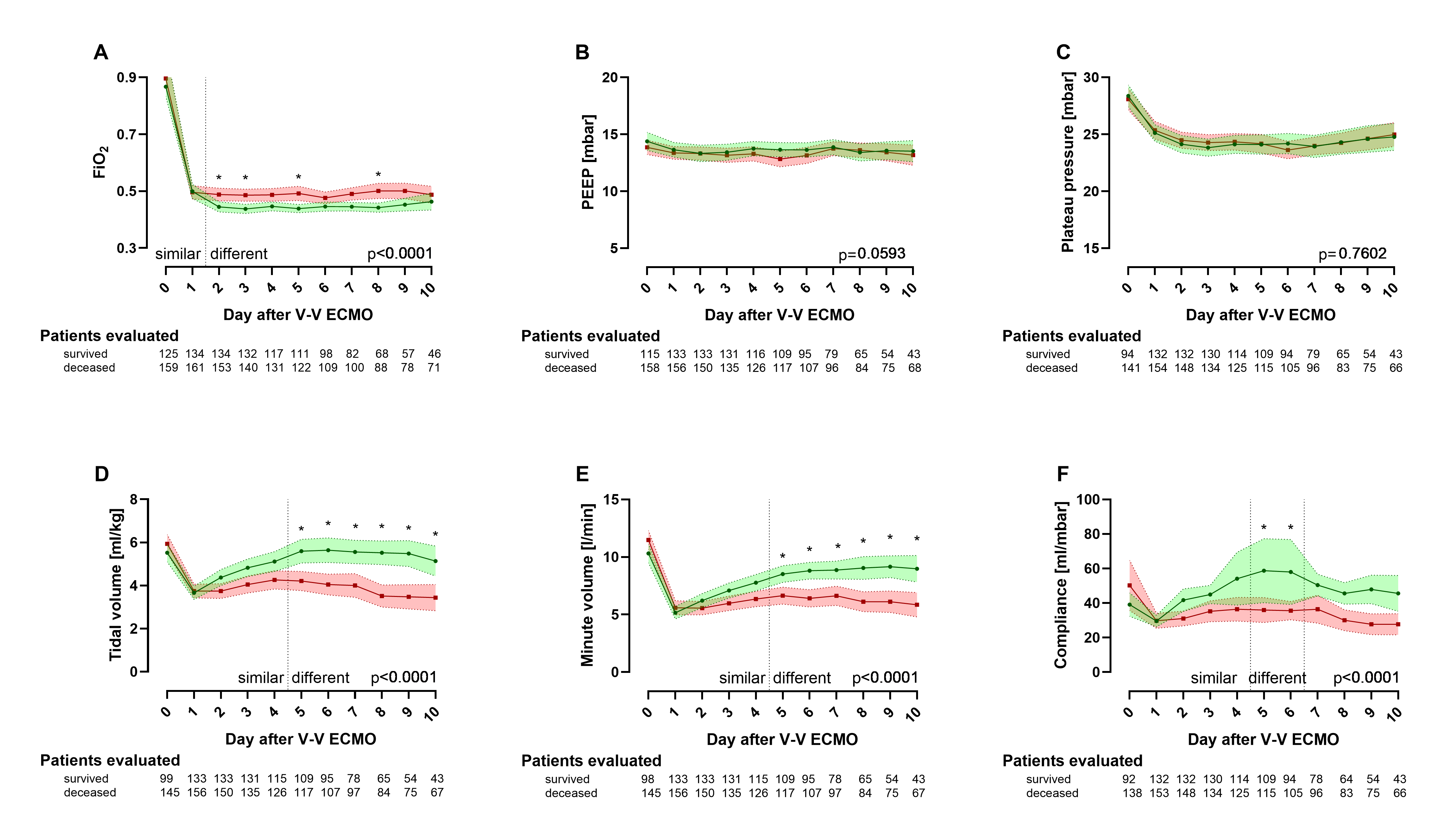


**Suppl. Figure 1: Ventilator settings in ARDS including only patients still on V-V ECMO.** All patients started V-V ECMO therapy on day 0. Green circles indicate hospital survival, red squares indicate deceased patients. P_group_ is shown in each figure. A: Ventilator FiO_2_, B: Ventilator PEEP, C: Ventilator plateau pressure, D: Tidal volumes, E: Minute volumes, F: Compliance. * marks significant post-hoc tests comparing groups on individual days. Abbreviations: FiO_2_ = inspiratory oxygen fraction, PEEP = positive end expiratory pressure, V-V ECMO = Veno-venous extracorporeal membrane oxygenation

**
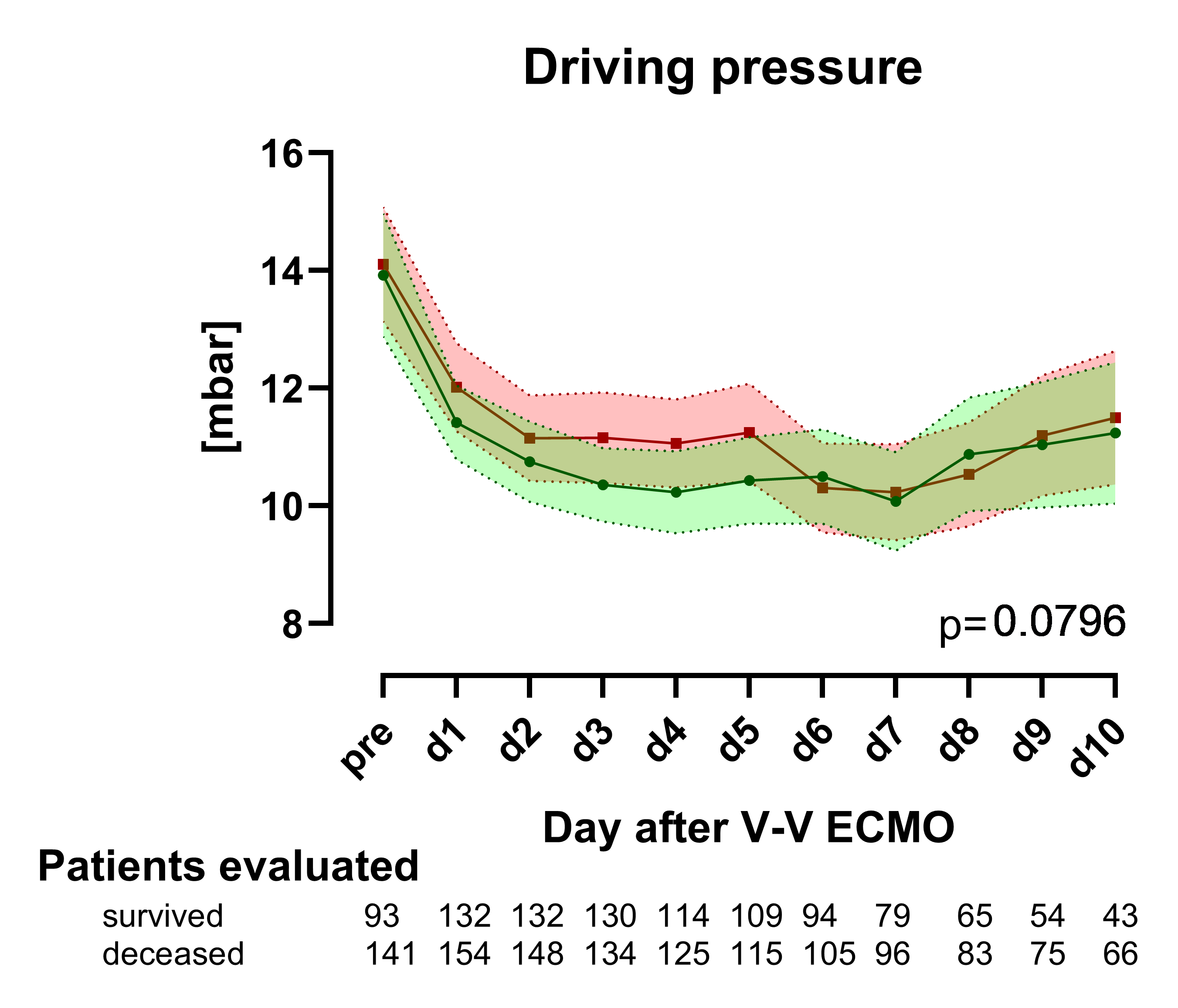
**

**Suppl. Figure 2: Driving pressure in ARDS including only patients still on V-V ECMO.** All patients started V-V ECMO therapy on day 0. Patients already weaned from ECMO are excluded. Green circles indicate hospital survival, red squares indicate deceased patients. P_group_ is shown in the figure. Abbreviations: V-V ECMO = veno-venous extracorporeal membrane oxygenation


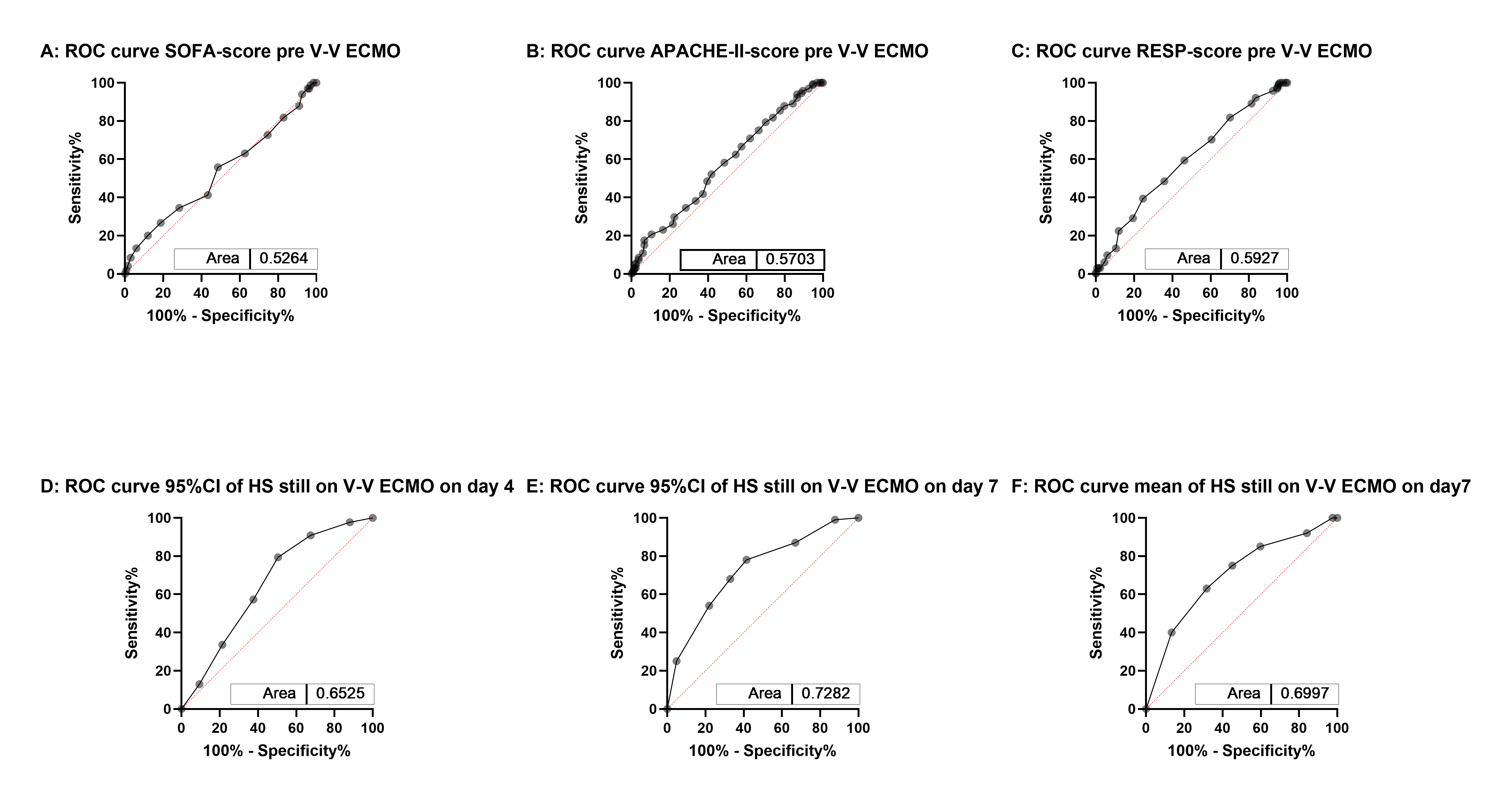


**Suppl. Figure 3: Receiver operating characteristic curves of different prediction models of hospital survival.** A: SOFA score pre ECMO had the lowest area under the curve (AUC), B: APACHE-II-score pre ECMO performed marginally better, C: RESP-Score pre ECMO performed best among all score available pre ECMO. D: scoring based on the 95% confidence interval (CI) was the first model showing an AUV >0.6, E: The highest AUC was achieved in our model of choice, F: Using the mean of survivors still on ECMO on day 7 could not match the area of our chosen model. Abbreviations: ROC = Receiver operating characteristic, V-V ECMO = veno-venous extracorporeal membrane oxygenation
